# A new Kirkpatrick–Baez-based scanning microscope for the Submicron Resolution X-ray Spectroscopy (SRX) beamline at NSLS-II

**DOI:** 10.1107/S1600577522007056

**Published:** 2022-07-29

**Authors:** E. Nazaretski, D. S. Coburn, W. Xu, J. Ma, H. Xu, R. Smith, X. Huang, Y. Yang, L. Huang, M. Idir, A. Kiss, Y. S. Chu

**Affiliations:** a National Synchrotron Light Source II, Brookhaven National Laboratory, Upton, NY 11973, USA; Bhabha Atomic Research Centre, India

**Keywords:** X-ray microscopy, KB optics, X-ray microscopy instrumentation

## Abstract

The development, construction, and first commissioning results of a new scanning microscope installed at the 5-ID Submicron Resolution X-ray Spectroscopy (SRX) beamline at NSLS-II are reported. The developed system utilizes Kirkpatrick–Baez mirrors for X-ray focusing. The instrument is designed to enable spectromicroscopy measurements in 2D and 3D with sub-200 nm spatial resolution. The present paper focuses on the design aspects, optical considerations, and specifics of the sample scanning stage, summarizing some of the initial commissioning results.

## Abstract 2

The development and initial commissioning of a new Kirkpatrick–Baez-based scanning microscope installed at the Submicron Resolution X-ray Spectroscopy beamline at NSLS-II are reported.

## 1. Introduction

The National Synchrotron Light Source II (NSLS-II) has a broad portfolio of imaging beamlines with multimodal and multiscale capabilities that comprise the Imaging and Microscopy Program. In particular, the Submicron Resolution X-ray Spectroscopy (SRX) beamline (Allwood *et al.*, 2018 ▸; Yadav *et al.*, 2017 ▸) plays a vital role in filling the resolution gaps between the microprobe beamlines such as the X-ray Fluorescence Microprobe (XFM) (Ding *et al.*, 2021 ▸), Tender Energy X-ray Absorption Spectroscopy (TES) (Northrup, 2019 ▸), and nanoscale imaging beamlines that include the Hard X-ray Nanoprobe (HXN) (Yan *et al.*, 2018 ▸; Nazaretski *et al.*, 2015 ▸, 2017 ▸), the Full-field X-ray Imaging (FXI) (Ge *et al.*, 2018 ▸; Coburn *et al.*, 2019 ▸), and the Coherent Diffraction Imaging (CDI) beamlines (Williams *et al.*, 2017 ▸). To effectively bridge the micro-imaging capabilities with the nanoscale imaging capabilities at the NSLS-II, a new scanning X-ray microscope with state-of-the-art capabilities has been designed, constructed, and is under commissioning at the SRX beamline.

The Kirkpatrick–Baez (KB) mirror system is an ideal X-ray focusing optics for the SRX beamline because it offers achromatic focusing property that is important for spectroscopic X-ray imaging. It also provides high-efficiency focusing that is useful for high-throughput imaging applications. The design concept for the SRX’s nano-KB system is to optimize the X-ray focusing at ∼300 nm with an option to reach a minimum spot size down to ∼100 nm enabling robust 2D and 3D imaging. Compared with other recently developed nano-KB systems (Somogyi *et al.*, 2015 ▸; Quinn *et al.*, 2021 ▸; Johansson *et al.*, 2021 ▸), the target resolution goal is modest, for two reasons. First, from a scientific standpoint, the SRX’s role at NSLS-II is to bridge the imaging capabilities between the microprobes and the nanoscale imaging beamlines. Consequently, there is no imperative reason to push the spatial resolution at the expense of the focused flux. Second, from a technical perspective, unlike the other latest nanoprobes equipped with KB mirror systems, the SRX beamline has the existing beamline infrastructure that is not well suited for deep nanoscale imaging [further details of the SRX source and optical layout are given by Chen-Wiegart *et al.* (2016 ▸)]. The SRX’s endstation is built on the storage ring experimental floor, lacking specialized vibration isolation and elaborate temperature stabilization in the experimental hutch, both of which are crucial environmental requirements for high-spatial-resolution nanoprobes. In addition, the source-to-optics distance in the horizontal direction (denoted as parameter *p* later in the text) is only 15.16 m, which makes it extremely challenging to achieve diffraction-limited focus size while ensuring reasonable working distance. Retaining a decent working distance has a profound impact on various *in situ* and *operando* experiments. Consequently, the SRX’s nano-KB system is designed to optimize the balance between the focused flux and resolution while providing robust imaging capabilities along with ease of sample exchange and high-throughput measurements.

The key instrument parameters for the developed nano-KB system are summarized in Table 1 ▸. The pre-focusing optical scheme creates a secondary source in the horizontal direction at *Z* = 50.64 m with a full width at half-maximum (FWHM) beam size of ∼50 µm, optimized through a slit-scan of the secondary source aperture. Then, the effective source size is set by the size of the secondary source aperture. We use the aperture size of 10 µm as the nominal value for evaluating other parameters that are affected by the effective source size. As commonly implemented at other nanoprobes (Yan *et al.*, 2018 ▸; Martínez-Criado *et al.*, 2016 ▸; Morawe *et al.*, 2015 ▸), the SRX beamline does not have active optical components in the vertical direction. Thus, the effective source in the vertical direction is the primary X-ray source produced by the in-vacuum undulator with a 22 mm period (IVU-22), which has a FWHM size of 17.6 µm at 10 keV. The nano-KB is designed with a *p* value of 15.16 m in the horizontal direction and 65.63 m in the vertical direction with a very limited adjustable range due to the short length of the experiment hutch where the endstation is located. Significant design attention has been given to choosing the *q* value or the focal length of the nano-KB system, measured from the center of the optics to the focal point. A smaller *q* value increases the demagnification factor, which is beneficial to achieve higher resolution, while a larger *q* value increases the working distance, favorable for robust scientific application. A critical high-level design requirement is to achieve a working distance of 70 mm, which is a good compromise, enabling various *in situ* experiments at the SRX beamline. The working distance requirement dictates not only the focal length of the KB mirrors but also constrains KB mirror lengths, which implicitly defines the numerical aperture (NA) for nanofocusing, coupled through the incidence beam angle (θ) to the mirror. A rhodium mirror coating with an incidence angle of 3 mrad is chosen to ensure a broad operating energy range from 5 keV to 20 keV while avoiding absorption edges, a necessary scientific requirement for robust spectroscopy measurements. The parameters in Table 1 ▸ result from the overall consideration of the focus size, focus flux, and working distance. It is important to note that the nominal operating mirror apertures (*i.e.* the size of the incidence beam accepted by the KB mirrors) are much larger than the transverse coherence lengths in both vertical and horizontal directions. Under the nominal operating condition, the KB mirrors are not expected to produce the diffraction-limited focus size unless the effective source sizes are significantly decreased. The FWHM contributions due to the geometric source demagnification are 86 nm (H) and 83 nm (V). It is important to point out that the focus broadening contribution due to the slope errors, 2·δθ·*q* (where δθ is the slope error), is smaller than the geometric contribution in the horizontal direction. In the vertical direction, the focus broadening due to the slope error is larger due to the larger focal length. The final designed focus size is estimated by performing a quadrature summation of the diffraction-limited focus size, the demagnified source size, and the slope error contribution.

## 2. Overview of the microscope

The development of the KB-based scanning microscope has been leveraged by the expertise and experience of NSLS-II staff acquired through the in-house developments of multiple X-ray microscopy instruments (Gao *et al.*, 2018 ▸; Nazaretski *et al.*, 2013 ▸, 2014 ▸; Kim *et al.*, 2013 ▸; Hwu *et al.*, 2013 ▸). Two new features have been incorporated into the developed system – the presence of an in-vacuum interferometer system to monitor the positions of individual KB mirrors, and the use of line-focusing interferometry to encode the position of the sample during tomographic measurements. Both of these features will be discussed in greater detail in the design sections of the manuscript.

**Figure 1:**
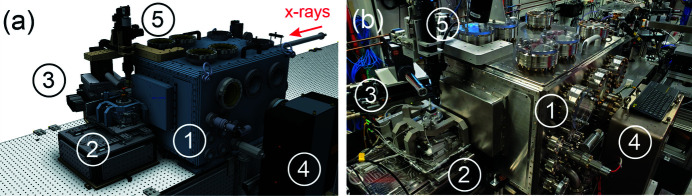
Overview of the KB-based scanning microscope installed at the SRX beamline. The key components are labeled. (*a*) CAD model and (*b*) photograph of the microscope. 1 – vacuum chamber housing the KB manipulation system; 2 – sample stage; 3 – fluorescence detector assembly; 4 – ion pump; 5 – visible-light microscope used for sample alignment.

An overview of the microscope is shown in Fig. 1 ▸. Fig. 1 ▸(*a*) depicts a computer-aided design (CAD) rendering of the system, and Fig. 1 ▸(*b*) shows the actual system installed in the experimental hutch of the beamline. The KB-optics are housed inside the vacuum chamber for thermal stability and to reduce radiation-induced contamination of the mirror surface. The optics is accessible for maintenance or repairs through the access doors and ports. The sample stage is placed outside the vacuum enclosure for flexibility of science experiments and easy sample exchange. The system is equipped with scanning and rotational capabilities, allowing for 2D imaging and 3D tomography experiments. The sections below describe the KB optics and its manipulation system, the sample stage, and present early commissioning results.

## 3. KB optics and manipulation system

**Figure 2:**
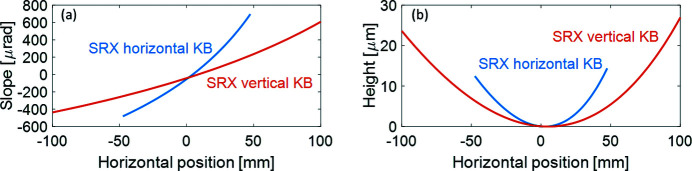
Measurement results of the SRX KB mirrors using the NSP instrument: (*a*) slope profiles, (*b*) height profiles, integrated using the slope measurement.

The two KB mirrors have been fabricated by JTEC Corporation and have been inspected in the NSLS-II Optical Metrology Laboratory. The NSLS-II Nano-accuracy Surface Profiler (NSP) (Qian & Idir, 2016 ▸) was used for characterizing the surface slope errors of the mirrors. The NSLS-II NSP instrument can accommodate large and heavy optical assemblies facing upwards or sideways and measure the surface errors in low to mid-spatial frequencies. In the NSP, an ELCOMAT3000/10 autocollimator coupled with a 2.5 mm-diameter aperture is used in the sample beam to scan the mirror surface, while an ELCOMAT3000/8 autocollimator is used in the reference beam to track the wobble of the air-bearing carriage. The purpose of the nano-metrology on the mirror surfaces included not only the measurement of the surface slope errors but also characterization of the induced surface error by the mirror holder, particularly for the vertical mirror. For the horizontal mirror, the effect of gravity is not important since it is acting in the transverse direction of the horizontal mirror. The measured slope profiles are presented in Fig. 2 ▸(*a*). The overall repeatability of the NSP measurements is better than 50 nrad RMS. Fig. 2 ▸(*b*) represents the surface height profiles of the KB mirrors obtained by integrating the measured slopes.

Although the scans are very repeatable, we only average those scans with repeatability better than 50 nrad RMS to avoid potential contributions from environmental perturbations and instabilities. In this way, the measurement repeatability after the averaging is less than the mirror slope error.

For the horizontal KB mirror, the measured slope profiles were fitted with its target ellipse by fitting the pitch of the metrology data and the beam center location on the mirror surface while keeping the source distance *p*, the image distance *q*, and the incidence angle θ fixed. Based on the non-linear least-square fitting result, the slope error of the horizontal KB mirror is 43 nrad RMS, which is less than the desired 50 nrad RMS as required by the design specifications.

**Figure 3:**
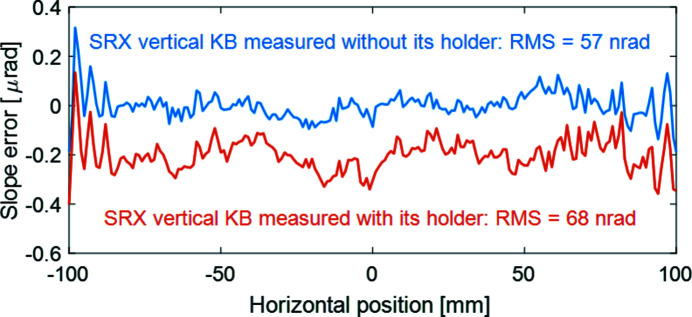
Residual slope errors of the vertical KB are evaluated with best ellipse fitting. The residual slope error is 57 nrad RMS when the mirror is measured without the mirror holder. When the mirror is measured with its holder, the residual slope error slightly increases to 68 nrad RMS. A vertical shift is added for better visualization and comparison.

For the vertical KB mirror, the least-square fitting included the pitch of the metrology data, the beam center location on the mirror surface, and the grazing-incidence angle θ, while the source distance *p* and the image distance *q* are fixed. The fitting residuals in Fig. 3 ▸ show that the vertical mirror also meets its tangential slope requirement (<70 nrad RMS), regardless of whether it is measured outside (57 nrad RMS) or inside (68 nrad RMS) its holder with a small change of the grazing angle θ (11 µrad without the holder or 6 µrad with the holder).

**Figure 4:**
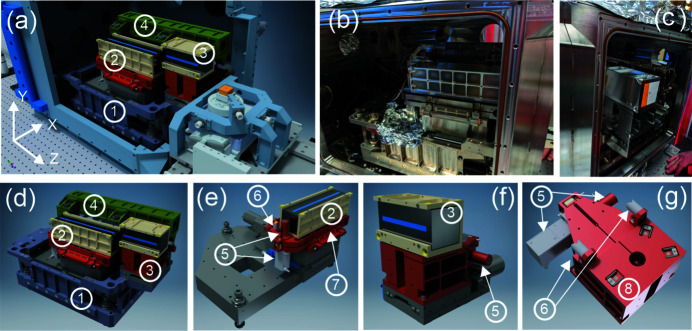
Overview of the KB manipulation system. Panels (*a*), (*d*), (*e*), (*f*), and (*g*) represent CAD models of various components. 1 – Invar base supporting both vertical and horizontal KB mirrors; 2 – vertical KB mirror assembly; 3 – horizontal KB mirror assembly; 4 – Invar reference frame for laser interferometer heads used to monitor/encode the position of the mirrors; 5 – coarse and fine actuators used to angularly align both mirrors; 6 – retroreflectors used in conjunction with the laser interferometer heads; 7 – flexure for the vertical KB mirror; 8 – flexure for the horizontal KB mirror. Panels (*b*) and (*c*) are the photographs of the installed system.

The developed KB-optics assembly is vacuum-compatible and resides inside the vacuum chamber (volume is ∼0.16 m^3^) mounted on a heavy granite slab (total weight is over 2 tons). The vacuum system consists of an ion pump (Gamma Vacuum TiTan 500 l) directly attached to the vacuum chamber and the maglev turbo-pump (Edwards Vacuum STP-L301) vibrationally decoupled from the ion pump. The roughing scroll pump (Edwards Vacuum XDS35i) is installed outside the experimental hutch on a vibration isolation pad to minimize ground vibrations. The system can be operated both in-vacuum and back-filled with He gas. Thin (50 µm-thick) diamond X-ray windows are mounted on removable flanges of the vacuum enclosure, with easy replacement capability. The vacuum chamber is decoupled from the KB-motion mechanics and is mounted directly on the granite slab through a bellows system. Both the KB-motion system and the vacuum chamber can be manually manipulated independently to ensure mutual alignment with respect to the incoming X-ray beam. The overall arrangement and the actual photographs of the KB vacuum chamber assembly are shown in Figs. 4 ▸(*a*)–4(*c*).

The entire KB-optics manipulation mechanism resides on an Invar-36 baseplate cradle assembly for improved thermal stability. Two independent motion stacks are employed to align the vertical and horizontal focusing mirrors. Figs. 4 ▸(*d*)–4(*f*) depict CAD models of the entire assembly along with the individual KB stacks. Detailed information about the motion degrees of freedom and motion parameters is summarized in Table 2 ▸.

**Figure 5:**
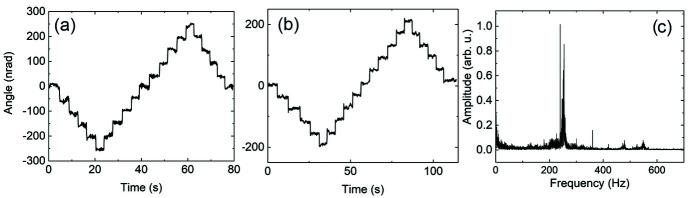
Characterization of the KB manipulation system. Panels (*a*) and (*b*) demonstrate 50 nrad angular motion of the horizontal and vertical mirrors, respectively. The angular motion has been verified by an external laser interferometer. Panel (*c*) demonstrates the fast Fourier transform spectrum of the vertical mirror flexure assembly. The fundamental resonance frequency is ∼250 Hz, making it less sensitive to ambient and cultural vibrations.

The electrical discharge machining (EDM) technique has been used to fabricate flexures for fine angular adjustment of both KB mirrors. In addition to the built-in optoelectronic encoders of the actuators, an external Invar reference frame mounted on a baseplate has been constructed. It accommodates independent interferometric sensors heads (Attocube FPS 3010) for proving external encoder signals for the actuators. Fig. 4 ▸(*g*) shows a pair of retroreflectors mounted on the flexures to provide angular pitch readings for both KB mirrors. Figs. 5 ▸(*a*) and 5 ▸(*b*) demonstrate the 50 nrad angular steps performed by the vertical and horizontal mirrors, respectively, measured by the interferometric sensors. The fine pitch motion for both mirrors can be operated using either the built-in native piezo encoder or the external interferometric sensors. In addition, the presence of a global reference frame provides valuable information regarding the long-term thermal drift of the optics due to thermal instabilities present in the experimental hutch. During the design phase of the instrument, significant consideration has been given to the stiffness aspects of the constructed system. Due to the large weight of the KB mirrors (∼2.5 kg is the weight of a vertical mirror), it is challenging to keep the resonance frequencies high and decoupled from the cultural vibrational noise. After several iterations and experimental validations, the final flexure structure has been constructed with a fundamental resonance frequency of ∼250 Hz, as shown in Fig. 5 ▸(*c*).

In summary, the thorough design and characterization of the KB optics along with its manipulation system ensure that required optical and mechanical characteristics are met within a given beamline layout, infrastructure, and cultural vibrational environment.

## 4. Sample stage

**Figure 6:**
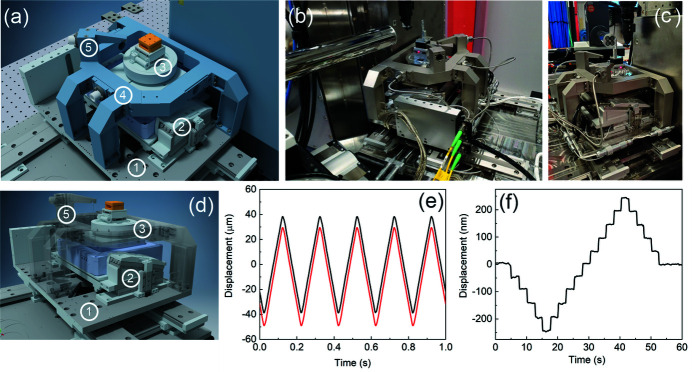
Overview and performance of the sample stage. Panels (*a*) and (*d*) show CAD models of the sample stage. The key components are enumerated: 1 – base long-travel-range *X*,*Z* stage; 2 – high-load vertical stage; 3 – scanner/rotary stage assembly; 4 – Invar reference frame; 5 – laser interferometry system. Panels (*b*) and (*c*) are photographs of the sample stage installed at the SRX beamline. Panel (*e*) shows a comparison between a 5 Hz raster scan encoded by the built-in scanner encoder (black line) and the external laser interferometer (red line). The interferometer readings are offset for clarity. Panel (*f*) shows a 50 nm step-scan performed at the beamline; the background noise level does not exceed 5 nm peak-to-peak amplitude.

The sample stage assembly is shown in Fig. 6 ▸, and Table 3 ▸ annotates all motions, actuators, resolutions, and respective travel ranges. During the design phase of the sample stage, a strong emphasis has been given to fast scanning and the ability to perform tomographic measurements. Due to geometrical limitations of the available space envelope, the use of high-quality large-form-factor air-bearing stages is not possible. Consequently, a much smaller, piezo-driven rotary stage has been implemented. It is well understood that the quality of the rotational motion in compact stages may not be sufficient for nano-tomography. Therefore, the sample stage is equipped with an external reference frame that accommodates the line-focusing laser-interferometry position-encoding system that tracks motions above the rotational stage and enables sample encoding during tomographic measurements. The base stage consists of low-profile, long-travel-range piezo-driven linear stages with a high-load wedge-based vertical stage. The base stage provides ‘coarse’ sample motion. High-resolution scanning is conducted using an *XYZ* scanner with a total travel range of 100 µm in each direction (nPoint NPXY100Z100-135), which is mounted on top of a vertical base stage. Both the scanner and the base stage have an aperture in the middle to enable proper cable management. The rotary stage is mounted and recessed inside the piezo-scanner. A high-precision diamond-turned reference disk with a circularity error of ∼50 nm and 70 mm in diameter is attached to the top of the rotary stage. The two linear piezo-stages are mounted on the reference ring and enable sample centering on the rotational axis. The maximum sample load supported by the assembly is ∼500 g. A JINPAT 30-leads slip-ring has been used to route the centering stages cables and enable infinite rotation. In addition, four electric contacts terminated with a Molex connector are available at the sample location and can be used for *in situ*/*operando* experiments. The native radial displacement errors of the rotary stage, measured at the sample location, are ∼2 µm. The line-focusing interferometry in the *X*, *Y*, and *Z* directions allows direct measurements of the sample displacement (right above rotation) during scanning experiments. In addition, it allows for tracking and potential compensation of rotational errors during 3D imaging experiments (Kim *et al.*, 2013 ▸). The Invar reference frame is mounted on the base stage, providing the relative positioning reference of the sample stage stack to the base stage. All intermediate connecting elements and adapters installed on the sample stage assembly are manufactured out of Invar-36 for improved long-term thermal stability. Two modes of sample scanning are available during the experiments. First, the native capacitive encoder can be used for sample scanning. Second, the feedback loop can be closed on the external interferometer and allows for more accurate (accounting for possible drifts) position encoding. Fig. 6 ▸(*e*) demonstrates a comparison between native and interferometric encodings. The two trajectories are offset for clarity. The 80 µm scan with 5 Hz mechanical frequency shows no distortion of the trajectory due to sample motion (with a 100 nm step size, the data acquisition rate equals 8 kHz). Fig. 6 ▸(*f*) demonstrates the 50 nm step scan performed by the sample stage and measured by the interferometer. Each step can be clearly resolved with the background peak-to-peak noise of ∼10 nm caused by instabilities present in the experimental hutch. A sound-absorbing enclosure to be installed around the sample stage in the near future may further decrease the background noise. Nevertheless, even in the present configuration, the sample noise levels are adequate for ∼100 nm resolution imaging experiments.

## 5. Early commissioning results

**Figure 7:**
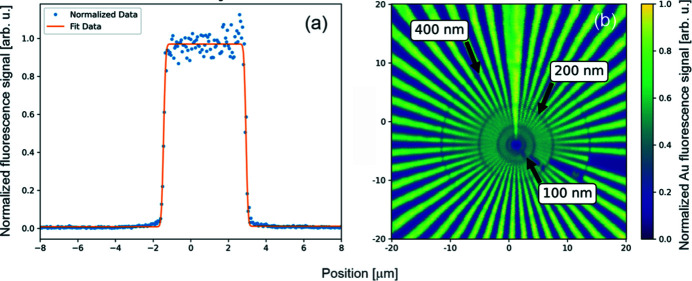
Initial X-ray commissioning results performed at the SRX beamline. The measurements were taken at 12 keV photon energy; the secondary source aperture has been closed down to 10 µm. Panel (*a*) demonstrates a horizontal line scan across a well defined line feature on the Siemen test pattern. The initial FWHM resolution obtained during the measurements is ∼188 nm during a scan with a 50 nm step-size, 20 ms dwell time and 16 µm travel range. Panel (*b*) depicts a 2D scan across a Siemens star test pattern. Sub-200 nm features in the horizontal direction are clearly visible. Scan parameters: 20 µm × 20 µm scan area, 50 nm step size, and 50 ms dwell time. The overall duration of the scan was ∼3 h.

Upon completion of assembly and testing, the microscope has been installed and surveyed in the experimental hutch of the SRX beamline. The new endstation has been installed downstream of the existing X-ray microscope and utilizes the detector infrastructure from the previous setup. The full-field detectors (Merlin from Quantum Detectors Ltd and a high-resolution X-ray camera using a scintillation screen) are used to check the quality of the incoming X-ray beam before and after the microscope. The on-axis visible-light microscope allows for a magnified view of the sample while letting the X-rays travel undisturbed through an aperture in the mirror. It is used for fast and reliable sample pre-alignment with respect to the incident X-ray beam. After the initial alignment of the KB mirrors, the well defined features in a Siemens star test pattern were used for focus optimization. Fig. 7 ▸(*a*) demonstrates a one-dimensional fluorescence scan across a Pt test structure. Scans in both vertical and horizontal directions are performed to ensure astigmatism-free point focus at the sample location. In the vertical direction the KB mirrors are imaging the undulator source, while in the horizontal direction the KB mirrors are imaging the secondary source. As commonly used for nanoprobes, a trade-off between focus size and focused flux is used as the decision criterion for the size of the secondary source aperture in the horizontal direction. In the vertical direction, the white-beam slits in the front-end are used to control the coherent mode of the X-ray beam. Fig. 7 ▸(*a*) represents a one-dimensional initial X-ray measurement that demonstrates the FWHM resolution of ∼188 nm in the horizontal direction. The two-dimensional scan of the Siemens star test pattern (Applied Nanotools Inc.) with the one-dimensional Gaussian fitting on the edges of the structures (fluorescence signal of the Au *L*-edge) is shown in Fig. 7 ▸(*b*). The data have been acquired in fly-scan mode with scan parameters listed in the figure caption. As shown in the figure, sub-200 nm features are clearly visible in the horizontal direction. In the vertical direction, the resolution is ∼227 nm. The focused flux was measured at 12 keV, and different secondary source openings yielded values between ∼5.95 × 10^9^ photon s^−1^ (secondary source aperture size 10 µm) and ∼3.3 × 10^10^ photon s^−1^ (secondary source aperture size 50 µm). Based on the early commissioning results, further optimization of the instrument together with the upstream beamline components are necessary to enhance the focusing performance. Specifically, the vibrational characteristics of the high-heat-load optical components with active cooling (*i.e.* white beam mirror and monochromator) need to be further analyzed to achieve better imaging resolution.

## 6. Conclusion and outlook

In conclusion, we have designed, constructed, and installed a new scanning microscope at the Submicron Resolution X-ray Spectroscopy beamline at NSLS-II. The detailed design considerations and technical details are reported in the manuscript. The thorough laboratory-based characterization of both X-ray optics and mechanical components is elucidated. The early commissioning results demonstrated sub-200 nm X-ray focusing, as a more comprehensive characterization is underway.

## Figures and Tables

**Table 1:** Summary of the SRX’s nano-KB mirror parameters Energy-dependent parameters such as the transverse coherence length and diffraction-limited focus size are evaluated at 10 keV. Under routine operation conditions, the horizontal source size, defined by the secondary source aperture, ranges from 100 to 10 µm. In the vertical direction, the KB mirrors operate on the primary X-ray source from the IVU-22 undulator. Except for the slope errors, all relevant parameters are based on FWHM values. The value in the slope error contribution column indicates the broadening of the focus size due to the slope errors.

Direction	Source size (µm)	*p* (m)	*q* (mm)	Active length (mm)	θ (mrad)	KB aperture (µm)	NA	Transverse coherence length (µm)	Slope errors(nrad)	Diffrac.-limited focus (nm)	Demag. focus (nm)	Slope error contribution (nm)	Final designed focus (nm)
Horizontal	10	15.16	130	110	3	330	1.27×10^−3^	94	50	49	86	30	103
Vertical	17.6	65.63	310	210	3	630	1.02×10^−3^	231	70	61	83	101	144

**Table 2:** Summary of motion actuators used in the KB-mirror assembly

Motion	Actuator	Range	Resolution
Vertical mirror, *Y*	Stepper motor	±5 mm	0.5 µm (full step)
VM pitch (coarse)	PI Nexline N-111	−0.5 to 4.5 mrad	∼220 nrad
VM pitch (fine)	PI P-841 piezo	±0.67 mrad	<50 nrad
Vertical mirror, *Z*	Manual	±5 mm	50 µm
			
Horizontal mirror, *X*	Stepper motor	±5 mm	1.0 µm (full step)
HM pitch (coarse)	PI Nexline N-111	−0.5 to 4.5 mrad	∼220 nrad
HM pitch (fine)	PI P-841 piezo	±1.5 mrad	<50 nrad

**Table 3:** Summary of all motions of the sample stage

Motion	Actuator		Range	Resolution
Coarse *X*, *Z*	SmarAct		100/120 mm	1 nm
Coarse *Y*	SmarAct		±7.5 mm	1 nm
Fine *X*, *Y*, *Z*	nPoint NPXYZ100		100 µm	1 nm
Rotation	SmarAct		Infinite	∼260 nrad
Sample centering, *X*, *Z*	SmarAct		±3.5 mm	1 nm
User-available electrical connections for *in situ* experiments		4		
Maximum sample weight		0.5 kg		
